# Rechallenge after anti-tuberculosis drug-induced liver injury in a high HIV prevalence cohort

**DOI:** 10.4102/sajhivmed.v23i1.1376

**Published:** 2022-06-14

**Authors:** Muhammed Shiraz Moosa, Gary Maartens, Hannah Gunter, Shaazia Allie, Mohamed F. Chughlay, Mashiko Setshedi, Sean Wasserman, David F. Stead, Karen Cohen

**Affiliations:** 1 Department of Medicine, New Somerset Hospital, Cape Town, South Africa; 2 Department of Medicine, Faculty of Health Sciences, University of Cape Town, Cape Town, South Africa; 3 Department of Medicine, Division of Clinical Pharmacology, Faculty of Health Sciences, University of Cape Town, Cape Town, South Africa; 4 Department of Medicine, Division of Gastroenterology, Faculty of Health Sciences, University of Cape Town, Cape Town, South Africa; 5 Department of Medicine, Division of Infectious Diseases, Faculty of Health Sciences, University of Cape Town, Cape Town, South Africa

**Keywords:** tuberculosis, anti-tuberculosis drugs, drug-induced liver injury, positive rechallenge, pyrazinamide, treatment interruption

## Abstract

**Background:**

There are limited data on the outcomes of rechallenge with anti-tuberculosis therapy (ATT) following anti-tuberculosis drug-induced liver injury (AT-DILI) in a high HIV prevalence setting.

**Objectives:**

To describe the outcomes of rechallenge with first-line ATT.

**Method:**

Hospitalised participants with AT-DILI who were enrolled into a randomised controlled trial of N-acetylcysteine in Cape Town, South Africa, were followed up until completion of ATT rechallenge. We described rechallenge outcomes, and identified associations with recurrence of liver injury on rechallenge (positive rechallenge).

**Results:**

Seventy-nine participants were rechallenged of whom 41 (52%) were female. Mean age was 37 years (standard deviation [s.d.] ±10). Sixty-eight (86%) were HIV-positive, of whom 34 (50%) were on antiretroviral therapy (ART) at time of AT-DILI presentation. Five participants had serious adverse reactions to an aminoglycoside included in the alternate ATT regimen given after first-line ATT interruption: acute kidney injury in three and hearing loss in two. The median time from first-line ATT interruption to start of first-line ATT rechallenge was 13 days (interquartile range [IQR]: 8–18 days). Antiretroviral therapy was interrupted for a median of 32 days (IQR: 17–58) among HIV-positive participants on ART before AT-DILI. Fourteen participants had positive rechallenge (18%). Positive rechallenge was associated with pyrazinamide rechallenge (*P* = 0.005), female sex (*P* = 0.039) and first episode of tuberculosis (TB) (*P* = 0.032).

**Conclusion:**

Rechallenge was successful in most of our cohort. Pyrazinamide rechallenge should be carefully considered.

## Introduction

Liver injury is the most frequent complication of first-line anti-tuberculosis therapy (ATT) with an estimated incidence of 2% – 28%.^1^ Following recovery from anti-tuberculosis drug-induced liver injury (AT-DILI), rechallenge with hepatotoxic first-line anti-tuberculosis drugs (rifampicin, isoniazid and, in some circumstances, pyrazinamide) is recommended because second-line ATT regimens are less effective, longer and more toxic.^2^ While awaiting resolution of liver injury, a background ATT regimen is given, typically consisting of ethambutol and at least two other second-line anti-tuberculosis drugs.

There is limited evidence on rechallenge following AT-DILI in populations with high prevalence of HIV coinfection. There is limited evidence on optimal background ATT regimens, optimal ATT rechallenge protocols, risk factors for positive rechallenge, anti-tuberculosis drugs most frequently implicated in positive rechallenge, and interruption and re-initiation of antiretroviral therapy (ART) in people living with HIV (PLHIV) who present with AT-DILI.

This study is nested within our randomised placebo-controlled trial of intravenous N-acetylcysteine (NAC) in the management of AT-DILI, which has previously been reported.^3^ We describe the characteristics, background ATT regimens (alternate ATT regimens initiated after first-line ATT interruption), rechallenge regimens, and outcomes of rechallenge in those participants who were rechallenged with ATT. Among HIV-positive participants, we explore the impact of AT-DILI and drug rechallenge on initiation or interruption of ART.

## Methods

### Study participants

Participants with AT-DILI admitted to three hospitals in Cape Town, South Africa, were enrolled in a pragmatic randomised placebo-controlled trial of intravenous NAC. Anti-tuberculosis drug-induced liver injury was defined as an alanine aminotransferase (ALT) ≥ 3 times the upper limit of normal if symptoms of hepatitis were present, or an ALT ≥ 5 times the upper limit of normal without symptoms of hepatitis.^4^ Other trial inclusion criteria were age 18 years or older, taking first-line therapy for tuberculosis (TB), and liver injury attributed to ATT.

After completion of the NAC or placebo infusion, decisions regarding clinical management were made by clinicians at participating hospitals and outpatient clinics. This included decisions regarding background ATT initiation and regimen, whether to rechallenge ATT, choice of rechallenge regimen, and interrupting, rechallenging, or initiating ART. Participants were followed up until the study primary endpoint (ALT reaching < 100 U/L) was reached and ATT rechallenge was completed. We included all trial participants who were rechallenged with at least one anti-tuberculosis drug in this analysis.

### Identification and assessment of positive rechallenge cases

‘Positive rechallenge’ is recurrence of liver injury on drug rechallenge. For this analysis, we defined positive rechallenge as doubling of ALT or total bilirubin concentration after rechallenge of an anti-tuberculosis drug.^5^ A multidisciplinary causality assessment panel including a clinical pharmacologist, a pharmacist, an infectious diseases specialist and a general physician assessed cases with a positive rechallenge, and identified the drug that was most likely to be causative, or any non-drug related cause for the increase in ALT or bilirubin.

### Statistical analysis

Categorical data were described using counts and percentages. Numerical data were described using means and standard deviations if normally distributed and medians and ranges if non-normally distributed. We compared parametric data using the Student’s *t*-test, non-parametric data using the Wilcoxon rank sum test and categorical data using the Fisher’s exact test. When comparing proportion with positive rechallenge between rechallenged drugs, we assumed that the three groups were independent. A *P*-value of < 0.05 was considered to be statistically significant throughout. Data were analysed using Stata (Version SE/15.1 Statacorp, College Station, Texas, United States).

We calculated ‘time from first-line ATT interruption to start of rechallenge’ as the interval from the date of AT-DILI presentation and first-line ATT discontinuation to the date that the first rechallenged drug was introduced. In HIV-positive participants on ART, we calculated ‘ART interruption time’ as the interval from the date of presentation with AT-DILI and ART discontinuation to the date of ARTre-initiation. In HIV-positive participants not on ART, we calculated ‘delay in ART initiation time’ as the interval from date of presentation with AT-DILI to the date of ART initiation.

### Ethical considerations

The study was conducted in accordance with the principles of the Declaration of Helsinki and the International Conference for Harmonisation.^6,7^ The study protocol was approved by University of Cape Town Human Research Ethics Committee and the Western Cape Department of Health (HREC 087/2012). Participants provided written informed consent. The trial was registered with the South African National Clinical Trials Registry (SANCTR: DOH-27-0414-4719).

## Results

**FIGURE 1:**
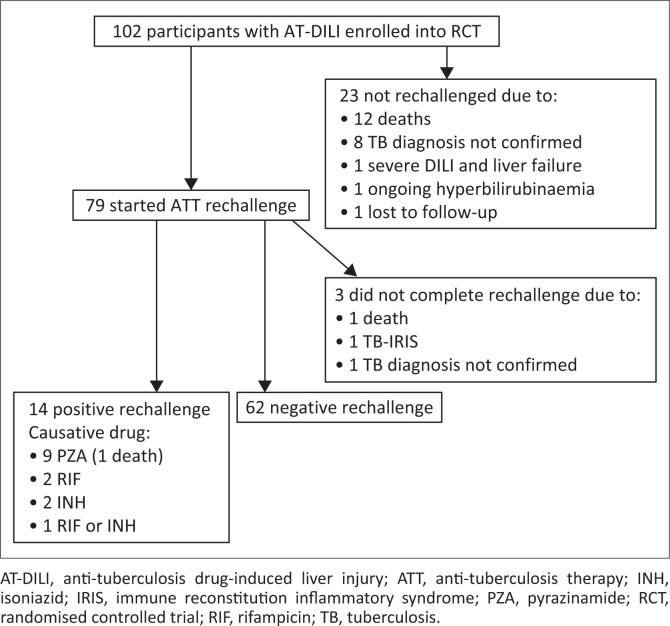
Anti-tuberculosis drug rechallenge following drug-induced liver injury in N-acetylcysteine randomised controlled trial participants.

Seventy-nine of 102 participants (77%) with AT-DILI enrolled into the randomised trial were rechallenged with ATT (Figure 1). Reasons for not rechallenging 23 participants were: 12 died before rechallenge was attempted, 8 had insufficient evidence of TB to justify rechallenge, 2 had prolonged hyperbilirubinaemia and were placed on second-line ATT because the clinical team deemed first-line ATT rechallenge to be unsafe, and 1 was lost to follow-up before planned rechallenge could be commenced.

Baseline characteristics of the 79 rechallenged participants, grouped by positive and negative rechallenge, are described in Table 1. Sixty-eight of the 79 (86%) participants rechallenged were HIV-positive, 34 of whom were on ART at presentation with AT-DILI, (27 on an efavirenz-based regimen and 7 on a lopinavir plus ritonavir-based regimen) and 18 were on cotrimoxazole prophylaxis.

**TABLE 1:** Baseline characteristics of participants with positive and negative rechallenge following anti-tuberculosis drug-induced liver injury.

Baseline characteristics	Positive rechallenge (*n* = 14)	Negative rechallenge (*n* = 65)	All rechallenged participants (*n* = 79)	*P* *
**Age (years)**				0.368
Mean ± s.d.	35 ± 12	38 ± 9	37 ± 10	
**Female**				0.039
*n*	11	30	41	
%	79	46	52	
**Weight (kg)**				0.418
Median	59	54	54	
IQR	50–74	46–64	47–64	
**First time on TB treatment**				0.032
*n*	14	47	61	
%	100	72	77	
**HIV-positive**				0.075
*n*	10	58	68	
%	71	89	86	
**CD4 count (cells/mm^3^) (for HIV-positive)†**				0.646
Median	56	76	70	
IQR	4–277	26–144	26–144	
**ALT (U/L)**				0.090
Median	255	385	357	
IQR	225–352	279–558	254–558	
**Total bilirubin (mmol/L)**				0.767
Median	44	49	47	
IQR	26–81	21–94	22–90	
**ALP (U/L)**				0.245
Median	126	183	175	
IQR	101–194	112–258	110–254	
**INR** ‡				0.288
Median	1.1	1.3	1.2	
IQR	1.0–2.1	1.1–1.8	1.1–1.8	
**Albumin g/L§**				0.758
Median	26	26	26	
IQR	19–35	21–30	21–30	

ALT, alanine transferase; ALP, alkaline phosphatase; INR, international normalised ratio; IQR, interquartile range; s.d., standard deviation; TB, tuberculosis.

* , Fisher’s exact test for categorical variables, *t*-test for parametric data, rank sum test for non-parametric data.

† , 26 with missing data.

‡ , 5 with missing data.

§ , 5 with missing data.

Forty-three participants commenced rechallenge during hospital admission, 10 of whom were referred to a community health centre to complete rechallenge. Twenty-five participants were rechallenged at a community health centre and 11 at a stepdown inpatient TB care facility.

Sixty-eight of 79 participants (86%) rechallenged were initiated on background ATT after first-line ATT interruption prior to rechallenge (Table 2). In the remaining 11 participants, background ATT was not commenced; reasons for this decision were not documented. All 68 participants initiated on background ATT received a fluoroquinolone, and 58 received an aminoglycoside (46 kanamycin, 11 amikacin, 1 streptomycin). Five of the participants who received an aminoglycoside (9%) had a serious adverse drug reaction: acute kidney injury in 3, and hearing loss in 2.

**TABLE 2:** Background anti-tuberculosis drug regimens prescribed following anti-tuberculosis drug-induced liver injury.

Background anti-tuberculosis drug regimen	Number of participants
Ethambutol + moxifloxacin + aminoglycoside†	54
Ethambutol + moxifloxacin +ethionamide	6
Ethambutol + moxifloxacin	4
Ethambutol + moxifloxacin + ethionamide + aminoglycoside‡	2
Moxifloxacin + ethionamide + aminoglycoside§	2
No background anti-tuberculosis therapy	11

† , 42 participants received kanamycin, 11 amikacin, 1 streptomycin.

‡ , Both participants received kanamycin.

§ , Both participants received kanamycin.

Most participants (96%) were rechallenged with a minimum of two individual drugs re-introduced sequentially and in full dosages (Table 3). Three participants completed only rifampicin rechallenge after which further rechallenge was discontinued: one was found to have no evidence of TB, one had worsening canalicular enzymes (likely due to TB-immune reconstitution inflammatory syndrome [IRIS] rather than AT-DILI recurrence), and one died from sepsis and multi-organ failure before completion of rechallenge.

**TABLE 3:** Sequence of anti-tuberculosis drug rechallenge.

Rechallenge regimen	Participants	
	*n*	%
RIF → INH → PZA†‡	38	50
INH → RIF → PZA	6	8
RIF → INH	26	30
INH → RIF	4	5
RIF → PZA	1	1
INH → PZA	1	1
RIF	3	5

INH, isoniazid; PZA, pyrazinamide; RIF, rifampicin.

† , One participant was rechallenged with RIF and INH concomitantly, followed by PZA.

‡ , One participant was rechallenged with RIF, PZA and INH concomitantly.

First-line anti-tuberculosis drugs were rechallenged at full dose. Drugs were rechallenged sequentially in 77 of 79 participants, with new drugs introduced at approximately 3-day intervals (Table 3). Rechallenge regimens differed in the sequence in which individual drugs were re-introduced: rechallenge commenced with rifampicin in 68 participants and with isoniazid in 11 (Table 3). The clinical care team elected not to rechallenge with pyrazinamide in 22 of 72 participants who had interrupted ATT due to liver injury during the intensive phase, because of the severity of the liver injury. The median time from first-line ATT interruption to start of rechallenge was 13 days (interquartile range [IQR]: 8–18 days).

### Positive rechallenge

There were 14 positive rechallenges in the 79 rechallenged participants (18%). Positive rechallenge was associated with female sex (Fisher’s exact test *P* = 0.039) and first episode of TB (Fisher’s exact test *P* = 0.032) (Table 1). The median time from first-line ATT interruption to start of rechallenge was similar between those with positive and negative rechallenge: median 12 days (IQR: 8–16 days) and 13 days (IQR: 9–18) respectively, Wilcoxon rank sum *P* = 0.719.

Rechallenge was positive in 9/46 participants rechallenged with pyrazinamide, 2/78 rechallenged with rifampicin, and 2/74 rechallenged with isoniazid. One participant had a positive rechallenge after sequential introduction of rifampicin and isoniazid. On causality assessment, both drugs were potentially implicated in the positive rechallenge because the participant’s serum ALT only settled after both drugs were withdrawn.

The proportion with a positive rechallenge was significantly higher among those rechallenged with pyrazinamide than among those rechallenged with rifampicin or isoniazid, Fisher’s exact test *P* = 0.005. One of the participants with positive pyrazinamide rechallenge developed a fatal systemic hypersensitivity reaction with rash, jaundice and acute kidney injury.

One participant had markedly increased serum canalicular liver enzymes (alkaline phosphatase and gamma-glutamyl transferase) at AT-DILI presentation which increased further after rifampicin rechallenge. The hospital clinicians assessed this as a positive rifampicin rechallenge and stopped rifampicin. However, the canalicular enzymes continued to increase after rifampicin cessation. On causality assessment, the increased canalicular enzymes were attributed to TB IRIS rather than a positive rifampicin rechallenge.

Antiretroviral therapy was interrupted at presentation with liver injury in 26 of the 34 (79%) HIV-positive participants who were receiving ART. At 8 weeks’ follow-up, 24 of these 26 participants had been re-initiated on ART: 21 recommenced their previous efavirenz-based regimen and three were switched from efavirenz-based to boosted protease inhibitor-based ART. The median ART interruption time was 32 days (IQR: 17–58). Twenty-one of 34 (62%) HIV-positive participants who were not on ART at the time of AT-DILI were initiated on ART after ATT rechallenge, after a median of 53 days (IQR: 35–91). Fifteen of 68 (22%) HIV-positive participants were not yet on ART when study follow-up ended.

## Discussion

In our cohort of patients with AT-DILI, the majority of whom had advanced HIV disease, rechallenge was attempted in the majority (77%). A wide variety of background regimens were used during rechallenge; adverse reactions to aminoglycosides in the background regimen were common. Rechallenge was positive in 18%, and was associated with female sex and first episode of TB. Positive rechallenge was significantly more common with pyrazinamide rechallenge than with isoniazid or rifampicin rechallenge. Positive rechallenge resulted in delays in initiating or commencing ART.

### Risk of positive rechallenge

In a recent network meta-analysis of ATT rechallenge regimens in participants with AT-DILI,^8^ 11% of those rechallenged with a sequential full dose regimen had a positive rechallenge. This is lower than the 18% we observed and could be explained by the longer rechallenge regimens used in the studies included in the meta-analysis. The majority of participants in the meta-analysis were rechallenged with rifampicin on day 1, isoniazid on day 8 and pyrazinamide on day 15–18, whereas the majority of our study participants were rechallenged with rifampicin on day 1, isoniazid on day 4 and pyrazinamide on day 7.

We found that women and participants with their first episode of TB were more likely to have a positive rechallenge. Other studies have also found female sex to be associated with AT-DILI^9,10^ as well as with positive ATT rechallenge.^11^ We did not find low serum albumin or increased age to be associated with positive rechallenge, in contrast to previous studies.^12,13^

### Pyrazinamide rechallenge

Pyrazinamide was the main cause of positive rechallenge in our study, with positive rechallenge in 20% of those rechallenged. Positive pyrazinamide rechallenge contributed to the death of one study participant. In a small randomised trial, 6 of 25 (24%) participants rechallenged with a concomitant full dose regimen including pyrazinamide had a positive rechallenge compared with 0 of 20 in the sequential full dose regimen group excluding pyrazinamide.^11^ American Thoracic Society guidelines advise against rechallenging pyrazinamide after severe AT-DILI.^4^ With increasing availability of effective second-line anti-tuberculosis drugs including fluroquinolones, linezolid and bedaquiline, avoidance of pyrazinamide rechallenge in all cases of AT-DILI should be considered.

### Antiretroviral therapy interruption and re-initiation

There is little published data on the impact of AT-DILI on ART in PLHIV. In our study, 79% of participants on ART at the time of AT-DILI presentation had their ART interrupted, with a median interruption of 32 days. Antiretroviral therapy interruptions may impact on efficacy of therapy and contribute to the emergence of antiretroviral resistance.^14^ Median delay from AT-DILI presentation to ART initiation in our cohort was 53 days, and 22% of the cohort were not yet on ART when study follow-up ended. Delays in initiation of ART in patients with advanced disease have previously been shown to increase mortality.^15^

### Study limitations

Our study has limitations. Although our study was nested within a randomised control trial, it is descriptive, and was not powered to identify risk factors for positive rechallenge. Study follow-up ended after rechallenge was complete, and we therefore could not quantify the impact of positive rechallenge on outcomes of ATT or ART. Our study cohort had a high prevalence of HIV, and the findings may not be generalisable to lower HIV prevalence settings.

## Conclusion

In this cohort of patients with AT-DILI, the majority of whom were HIV-positive, pyrazinamide was the most common cause of positive rechallenge. Positive rechallenge resulted in delays in initiating or recommencing ART. Use of second-line anti-tuberculosis drugs should be considered as an alternative to pyrazinamide rechallenge.
